# Methotrexate induces production of IL-1 and IL-6 in the monocytic cell line U937

**DOI:** 10.1186/ar4444

**Published:** 2014-01-20

**Authors:** Nancy J Olsen, Charles F Spurlock, Thomas M Aune

**Affiliations:** 1 Division of Rheumatology, Department of Medicine, Penn State MS Hershey Medical Center, 500 University Drive, Hershey, PA 17033, USA; 2 Division of Rheumatology, Department of Medicine and Department of Pathology, Microgiology and Immunology, Vanderbilt University Medical Center, 1211 Medical Center Drive, Nashville, TN 37232, USA

## Abstract

**Introduction:**

Methotrexate (MTX) has been for decades a standard treatment in a wide range of conditions, from malignancies to rheumatoid arthritis (RA). Despite this long experience, the mechanisms of action of MTX remain incompletely understood. Reported immunologic effects of MTX include induction of increased production of some cytokines, an effect that seems to be at odds with the generally anti-inflammatory effects of this drug in diseases like RA. To further elucidate these immune activities, we examined effects of MTX on the human monocytic cell line U937.

**Methods:**

The U937 cell line was treated *in vitro* with pharmacologic-range concentrations of MTX and effects on production of interleukin (IL)-1, IL-6 and TNF alpha were measured. Changes in gene expression for IL-1 and IL-6 and specificities in the Jun-N-terminal kinase (JNK) signaling pathway including JNK 1, JNK2, JUN and FOS were also determined. The contribution of NF-kB, folate and adenosine pathways to the observed effects was determined by adding appropriate inhibitors to the MTX cultures.

**Results:**

MTX mediated a dose-dependent increase in IL-1 and IL-6 in U937 cells, as measured by secreted proteins and levels of gene expression. The increased cytokine expression was inhibited by addition of parthenolide and folinic acid, but not by caffeine and theophylline, suggesting that NF-kB and folates, but not adenosine, were involved in mediating the observed effects. When U937 cells were cultured with MTX, upregulated expression of JUN and FOS, but not JNK 1 or 2, also was observed.

**Conclusions:**

MTX induces expression of proinflammatory cytokines in U937 monocytic cells. These effects might mediate the known toxicities of MTX including pneumonitis, mucositis and decreased bone mineral density.

## Introduction

Methotrexate (MTX) was first introduced into clinical practice as a chemotherapeutic agent more than six decades ago and the continued use of this older drug is evidence of its remarkable therapeutic effects [1-3]. Treatment with MTX is a key reason for the transformation of acute childhood leukemias from being uniformly fatal to having long-term survival rates of 70% or more. In rheumatoid arthritis (RA), MTX is considered a cornerstone of all therapies and its use is largely responsible for improved functional and structural outcomes in these patients [4]. Even the newer biologic agents in RA are generally given in combination with MTX to maximize therapeutic effects [5]. At the same time, the adverse events profile of MTX requires application of caution in its use. Pulmonary toxicity with MTX is a rare but potentially fatal disorder and decreases in bone density may be another long-term consequence of treatment, especially at higher doses used in chemotherapeutic regimens [6].

The mechanisms of action of MTX are related at least in part to antiproliferative effects that are dependent on inhibition of dihydrofolate reductase and inhibition of nucleotide synthesis pathways [7]. Other activities, including release of adenosine and inhibition of polyamines, are thought to contribute to the anti-inflammatory effects [8]. Some actions of MTX also depend on stimulation of the production of reactive oxygen species (ROS) and induction of T lymphocyte apoptosis [9]. In previous studies, we have shown that MTX treatment of patients with RA restores toward normal expressed levels of genes and associated proteins related to cell cycle checkpoint pathways [10], and more recent studies suggest that expressed levels of genes related to folate metabolism also may be altered *in vivo* by MTX [11].

The many documented effects of MTX include stimulation of both pro- and anti-inflammatory pathways. In some situations, such as pneumonitis and mucositis, enhanced cytokine production has been postulated to contribute to tissue damage [12-14]. The loss of bone density seen after long-term treatment with MTX, which is a greater concern in chemotherapeutic than in anti-inflammatory regimens, also has been attributed to increased levels of cytokines and activation of nuclear factor kappa B (NF-kB) [15].

Our previous studies with MTX have been focused on effects in T lymphocytes, and have shown that these cells are primed by MTX for apoptosis by a JNK-dependent mechanism [10,16]. The objective of the present study was to examine effects of MTX on cells of monocyte lineage, utilizing the human line U937. Comparisons were made with another drug used for RA, hydroxychloroquine (HCQ), which acts through lysosomes including those in macrophages [17]. The findings of the present investigation show that MTX enhances production of the inflammatory mediators IL-6 and IL-1. Whether this action of MTX contributes to some of the effects of this drug *in vivo* in treated patients is discussed.

## Methods

### Materials

Methotrexate (MTX), hydroxychloroquine (HCQ), lipopolysaccharide (LPS), caffeine (CAFF), theophylline (THEO), folinic acid (FA) and parthenolide (PAR) were from Sigma-Aldrich (St Louis, MO, USA). Secreted interleukin (IL)-1beta, IL-6 and tumor necrosis factor (TNF)-alpha in culture supernatants were quantitated using enzyme-linked immunosorbent assay (ELISA) kits from Abcam (Cambridge, MA, USA) and results were expressed in standardized concentrations using reagents provided with these kits.

### Cell preparation and culture

The human cell lines U937 (monocytes) and Jurkat (T lymphocytes) were obtained from ATCC (Manassas, VA, USA) and were maintained in suspension culture with RPMI 1640 supplemented with 10% fetal calf serum (FCS). Cell viability was determined by trypan blue dye exclusion and by the Vybrant MTT Cell Proliferation Assay (Invitrogen, Carlsbad, CA, USA). MTT is 3-(4,5-dimethylthiazol-2-yl)-2,5 diphenyl tetrazolium bromide and is used to quantify numbers of cells in culture [18]. Concentrations of MTX, HCQ, LPS and PAR ranged from 0.01 uM to 1 uM, as indicated in individual experiments. Of note, the concentration of MTX achievable after oral ingestion of a 20 mg tablet yields a plasma concentration of 0.1 uM after 10 hours [10]. Cultures were incubated in a humidified atmosphere with 5% CO2 for 24 to 72 hours, as indicated in specific experiments. These studies were carried out in a human cell line and no institutional ethics approval or patient consent was required.

### Quantitative RT-PCR

Total RNA was purified from cell pellets using the Qiagen RNeasy Mini Kit (Germantown, MD, USA) and quantitated with a NanoDrop 2000 (Thermo Scientific, Wilmington, DE, USA). Preparation of cDNA was done using the High Capacity RNA-to-cDNA Kit (Applied Biosystems/Life Technologies, Carlsbad, CA, USA) with 100 to 200 ng RNA per synthesis reaction. RT-PCR analysis was performed for selected genes using TaqMan Gene Expression Assays (Life Technologies) with GAPDH as the housekeeping control gene with an ABI-7300 Real Time PCR instrument. Expression values are normalized to GAPDH levels using the following formula: 2^(GAPDH Ct-Test gene CT)^.

### Flow cytometry

Cells were suspended at 1 × 10^6^/ml in phosphate-buffered saline with 2% bovine serum albumin (BSA) and 0.1% sodium azide and surface stained with PE-Cy-7-labeled anti-CD14 (Becton Dickinson, Franklin Lakes, NJ, USA). Apoptosis was quantitated using the PE Annexin V Apoptosis Detection Kit (BD Pharmingen, BD Biosciences). This kit utilizes double-staining with PE Annexin V and 7-amino actinomycin D (7-AAD) to distinguish between viable cells and those that are undergoing apoptosis. Staining data were collected in a BD FACSCanto II in the Hershey Medical Center Flow Cytometry Core and FlowJo Software (Tree Star, Inc., Ashland, OR, USA) was used to analyze the results.

### Statistics

Data were expressed as mean and standard deviation of the mean. The significance between the two experimental groups was determined by Student’s *t* test or, for comparison of three or more groups, by a one-way analysis of variance (ANOVA) with Tukey’s or Dunnett’s multiple comparisons tests used as post tests where indicated. The analyses and the graphics were done with Prism 6.0 (GraphPad Software, Inc., La Jolla, CA, USA). *P* values of less than 0.05 were considered significant.

## Results

### Cell viability and apoptosis

**Figure 1:**
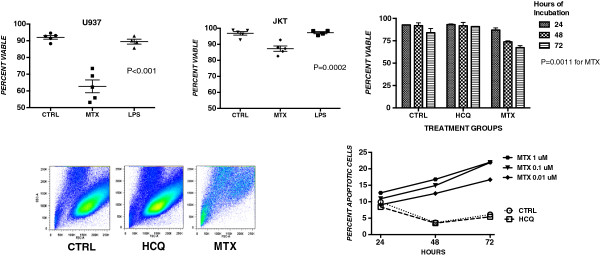
**U937 and JKT cell lines were cultured with or without MTX (1 uM) and LPS (25 ug/ml).** Viability determined by trypan blue dye exclusion was decreased by MTX in U937 but not JKT cell lines (top left). Decreased viability of U937 cells increased over time of incubation with MTX (1 uM) but not with HCQ (1 uM; top right). Forward/side scatter plots show changes consistent with decreased viability of U937 cells after 72 hours of culture with MTX (1 uM) but not with HCQ (1 uM; lower left). Apoptosis measured by 7-AAD was increased in dose- and time-dependent fashion by MTX but was not induced by HCQ (lower right). *P* values were determined by one-way ANOVA. 7-AAD, 7-amino actinomycin D; ANOVA, analysis of variance; HCQ, hydroxychloroquine; LPS, lipopolysaccharide; MTX, methotrexate.

We have shown previously that MTX primes Jurkat T cells for increased sensitivity to apoptosis and low levels of apoptosis are induced in these cells by MTX itself [10]. Compared to Jurkat T cells, U937 cells had reduced viability *in vitro* as measured by trypan blue dye exclusion (Figure 1). After 72 hours in culture, this difference was observed for cultures even with no added agents (92% vs. 96%; *P* = 0.001), with 1 uM MTX (63% vs. 87%; *P* = 0.0004) and 25 ug/ml LPS (89% vs. 97%; *P* = 0.0031). HCQ (1 uM) had no significant effect. A greater than 40% decrease in viable U937 cells in 48 and 72 hour MTX (1 uM) cultures was also measured using the MTT assay (*P* <0.002). Flow cytometry profiles confirmed the presence of fewer viable cells in the forward scatter/side scatter (FSC/SSC) window for the cultures with added MTX, but not with HCQ (Figure 1). MTX mediated a dose- and time-dependent increase in apoptosis of U937 cells as measured by 7-AAD (Figure 1); HCQ did not show an effect on the apoptosis profile.

### Cytokine expression

**Figure 2:**
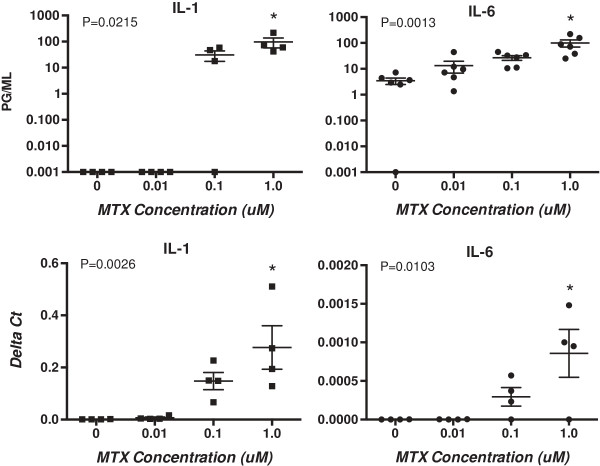
**Production of IL-1 and IL-6 measured by ELISA in supernatants of U937 cells after 72 hours of culture with MTX showed dose-dependent increases (top panels).** Similar increases in corresponding gene expression levels for IL-1 and Il-6, expressed as Delta Ct values, also were measured (bottom panels). *P* values were determined by one-way ANOVA and *post hoc* analyses confirmed significant (*P* <0.05) increases at MTX 1.0 uM (indicated by asterisks) compared to cultures with no added MTX for all four measures. ANOVA, analysis of variance; ELISA, enzyme-linked immunosorbent assay; IL, interleukin; MTX, methotrexate.

Secretion of both IL-1 and IL-6 into culture supernatants was increased by MTX in a dose-dependent fashion (Figure 2, top panels). The increases were significantly different from the control baseline at a MTX concentration of 1.0 uM. Expression levels of associated gene specificities for IL-1 and IL-6, normalized to GAPDH expression, were also significantly elevated in MTX cultures at the highest concentration tested, 1.0 uM (Figure 2 bottom panels). Levels of secreted TNF-alpha were also increased in 72 hour cultures from 13 pg/mL in control cultures to 408 pg/mL in MTX (1 uM) cultures (*P* = 0.022; not shown in figures).

### Mechanisms of cytokine induction

**Figure 3:**
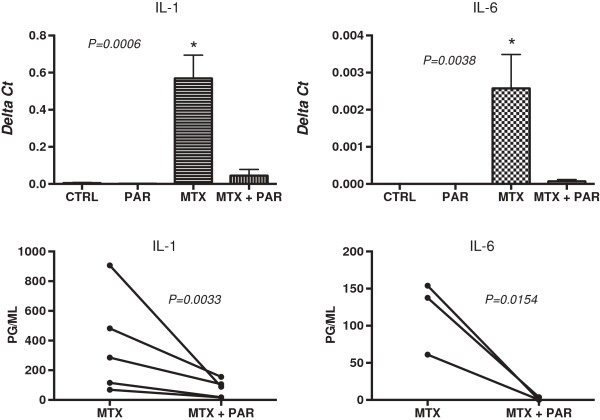
**IL-1 and IL-6 gene expression levels, normalized to GAPDH and expressed as Delta Ct values, were significantly increased by addition of MTX (1 uM) to 72 hour cultures of U937 cells and these increases were blocked by co-culture of MTX with PAR (1 uM) to levels that were not significantly different than those in control (CTRL) cultures (top two panels).***P* values for the gene expression comparisons were calculated by one-way ANOVA, and Dunnett’s post test showed that only the MTX cultures were significantly different (*P <0.05) from corresponding CTRL cultures. Furthermore, secreted IL-1 and IL-6 cytokines measured by ELISA in these 72 hour U937 supernatants were significantly decreased when cells were co-cultured with MTX and PAR (bottom two panels). Significance (*P* values) for the IL-1 and IL-6 supernatants was calculated by paired *t* test. ANOVA, analysis of variance; ELISA, enzyme-linked immunosorbent assay; IL, interleukin; MTX, methotrexate; PAR, parthenolide.

The role of NF-kB signaling in the effects of MTX on cytokine induction was investigated by adding the inhibitor PAR. Direct addition of PAR (1 ug/ml) to U937 cells had no effect on U937 viability, which was greater than 90% after 72 hours of culture. Co-culture of PAR with MTX resulted in significant decreases in IL-1 and IL-6 gene expression to levels that were not significantly different than control cultures (Figure 3, top panels). In addition, secreted levels of IL-1 and IL-6 were significantly decreased with addition of PAR to the MTX cultures (Figure 3 bottom panels). These results suggest that the MTX-induced upregulation of IL-1 and IL-6 is dependent on NF-kB.

**Figure 4:**
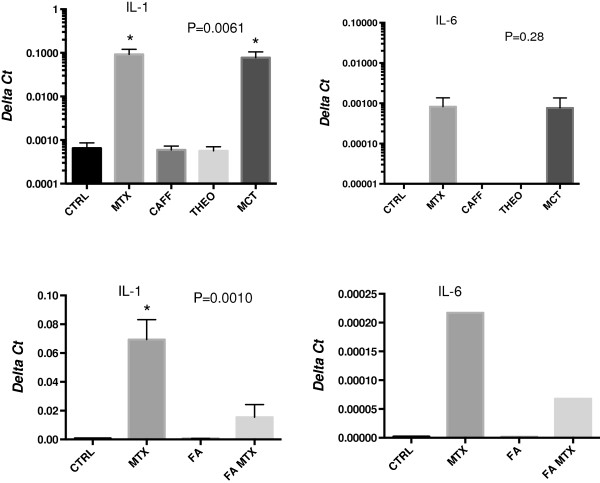
**IL-1 and IL-6 gene expression levels, normalized to GAPDH and expressed as Delta Ct values, were increased with MTX and not affected by addition of either CAFF or THEO (1 uM each).** Addition of CAFF and THEO along with MTX (MCT) did not block the stimulatory effects of MTX on IL-1 expression (top left). Significant differences for IL-1 gene expression were detected by one-way ANOVA (*P* = 0.0061) followed by Dunnett’s multiple comparisons test, comparing each condition to control (CTRL). Only MTX and MCT cultures (indicated by asterisks) were significantly different from CTRL (*P* <0.05). A similar pattern was observed for IL-6 expression (top right). Although the ANOVA result was not statistically significant, only MTX and MCT cultures had any detectable IL-6 expression. Co-culture of MTX with FA (1 uM) blocked significantly the normalized expression of IL-1 (bottom left), as shown by ANOVA (*P* = 0.0010) and in *post hoc* analyses only the MTX culture was significantly different from CTRL (*P <0.05). A similar pattern was observed in a single experiment for IL-6 expression (bottom right). ANOVA, analysis of variance; CAFF, caffeine; FA, folinic acid; IL, interleukin; MTX, methotrexate; PAR, parthenolide; THEO, theophylline.

Possible involvement of the adenosine pathway on these cytokine effects was probed by culturing U937 cells with THEO and CAFF [19]. These two drugs when added individually (1 uM) had no effect on cell viability. When present in culture in combination with MTX, no significant change in IL-1beta or IL-6 gene expression was observed (Figure 4, top panels), suggesting that the adenosine pathway was not responsible for the cytokine response. In contrast, addition of folinic acid to MTX cultures resulted in decreased IL-1beta and IL-6 gene expression (Figure 4, bottom panels) suggesting a role for folate-dependent pathways in mediating cytokine induction.

### Effects on JUN pathway genes

**Figure 5:**
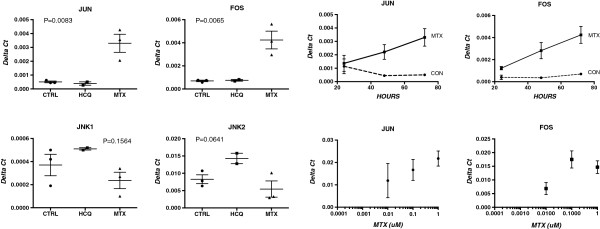
**Normalized gene expression (Delta Ct) values for JUN and FOS in U937 cells were increased with MTX (1 uM) but not with HCQ (1 uM).** The increases in JUN and FOS observed with added MTX were time- and dose-dependent (right two panels). Values represent mean and SEM; *P* values calculated using one-way ANOVA. ANOVA, analysis of variance; HCQ, hydroxychloroquine; MTX, methotrexate.

In previous studies we have shown that levels of JNK1 and JNK2 are decreased in lymphocytes from patients with RA, and that MTX treatment results in increased levels of these signaling molecules along with a decrease in sensitivity of lymphocytes to apoptotic signals [10]. To evaluate the role of these pathways in the observed U937 responses, we measured gene expression levels in cultured cells, and found that JUN and FOS, but not JNK 1 or JNK 2, were upregulated by MTX, but not by HCQ, in a time- and dose-dependent manner (Figure 5). Addition of PAR to these MTX cultures did not significantly decrease the levels of FOS and JUN (data not shown). Expression levels of JUN and FOS were each correlated with levels of IL-1beta gene expression (R^2^ values of 0.86 and 0.97, respectively).

## Discussion

The findings reported here demonstrate proinflammatory effects of MTX on human monocyte/macrophage cells including gene upregulation and secretion of the cytokines IL-1, IL-6 and TNF-alpha. The underlying mechanism appears to be consistent with an action on the NF-kB pathway rather than through adenosine receptors. Doses of MTX used in these studies are in a range that would be achievable with *in vivo* treatment of malignancies or autoimmune disease. So even though these are *in vitro* studies on a cell line, the results may have implications for actions of MTX in treated patients. Although no effects were observed on human peripheral blood cells (data not shown), localized tissue effects may contribute to some of the off-target actions of this drug.

These proinflammatory effects of MTX are of interest because this drug is widely used to treat inflammatory and autoimmune disorders including RA, psoriatic arthritis and inflammatory myopathies. Mechanisms by which the low-dose intermittent regimen has clinical effects in these diseases remain somewhat obscure. The earliest concept, borrowed from oncology applications, was that of anti-proliferative actions, thereby reducing the burden of inflammatory cells [7]. Other potential mechanisms have been proposed, including interactions with adenosine signaling pathways and generation of ROS [8,19,20]. In previous studies we have shown that MTX primes T cells for apoptosis, an action that is dependent on JNK signaling pathways [16]. Overall, these effects likely result in a reduced inflammatory burden that translates into decreased levels of damage in treated patients.

However, other effects of MTX that have been reported appear to be directly contradictory to those that would be desirable for treatment of inflammatory conditions like RA. It is possible that these responses are relevant to some of the toxic, or off-target, effects of MTX that include bone loss, mucositis and pulmonary inflammation, especially at the higher doses used in chemotherapeutic regimens. Consistent with this is a short-term chemotherapy model in which MTX treatment in rats resulted in activation of NF-kB and increases in plasma levels of IL-6 and TNF-alpha [15]. Other studies using MTX treatment in rats have shown induction of TNF-alpha, IL-1beta and macrophage inflammatory protein (MIP)-2 in the small intestine and these inflammatory cytokines likely mediate mucositis in the intestine and elsewhere in the gastrointestinal tract [12,14]. Relevant to pulmonary toxicity is the finding MTX has been shown to enhance expression of IL-1beta and Il-8 in a human bronchial cell line via the p38 MAPK signaling pathway [21]. Increased levels of these cytokines in localized areas of bone might explain why high doses of MTX have been associated with bone loss in oncology patients, although this has been rarely reported in patients treated with low-dose regimens for diseases like RA [22,23].

Although it might be anticipated or assumed that MTX would have negative regulatory effects on cytokine production, this in fact has not been clearly demonstrated in experimental models or in treated patients. An older investigation carried out prior to the availability of immunoassays failed to show inhibition of IL-1 secretion, although functional activity of the cytokine was reduced [24]. It has been suggested that levels of IL-1 in the joint space of patients treated with MTX may be decreased due to changes in local production or composition of synovial cell populations; but changes in peripheral blood were not shown [25]. Investigations into effects of MTX on IL-6 have had similar mixed results. In the murine glucose-6-phosphate isomerase-induced arthritis model, for example, treatment with MTX does not result in decreases in either IL-6 or TNF alpha [26]. In a study of osteoblasts, MTX alone had no effect on IL-6 synthesis, but it was able to mediate decreased IL-6 production by these cells in response to other inflammatory mediators [27]. This result suggests that the existing inflammatory milieu may impact cellular responses to MTX. Other findings suggest that the anti-inflammatory cytokine IL-10 may be induced along with proinflammatory mediators, and perhaps the relative balance varies to impact the ultimate physiological effect [13].

A limitation of our studies is that they were carried out in a cell line, and we did not observe stimulation of cytokine production in human peripheral blood mononuclear cells cultured with MTX (data not shown). However, monocyte lineage cells are only a small component of peripheral blood, and it is possible that effects of MTX on minor cell types, perhaps even subsets of circulating monocytes, are not sufficient to be measured in mixed cell populations. The concept that at least some cell types respond to MTX by activation of inflammatory pathways is consistent with the known adverse events of this treatment. It is possible that specialized monocyte/macrophages in tissue locations such as bone or mucosa might be more likely to generate inflammatory responses than cells in the circulation. Some manifestations in treated patients, including mucositis, are often tempered or blocked by the addition of folic acid supplements, which is consistent with the observed reversibility of the cytokine response with folinic acid. It is also possible that this is a dose-related effect of MTX and that the higher doses used in chemotherapeutic regimens are more likely to stimulate inflammatory pathways.

Another implication of the current findings is that if MTX stimulates production of even low levels of proinflammatory cytokines, this may be a reason why combining MTX treatment with cytokine blocker drugs is efficacious and has longer duration of drug survival than monotherapy treatments, at least in some patients [5,28]. Further studies to identify patients in whom this effect is significant could be useful to predict those who are more likely to benefit from addition of anti-cytokine agents to MTX.

## Conclusions

MTX upregulates in the monocyte cell line U937 the production of the proinflammatory cytokines IL-1, IL-6 and TNF alpha. The folate pathway is implicated in this response, while the adenosine signaling pathway is probably not involved. These results may have implications for explaining mechanisms of some off-target actions of MTX such as mucositis and pneumonitis as well as decreased bone density in oncology patients. Identification of patients in whom this response is significant might be useful in predicting the need for combination therapy with anti-cytokine agents.

## Abbreviations

7-AAD: 7-amino actinomycin D; ANOVA: Analysis of variance; BSA: Bovine serum albumin; CAFF: Caffeine; ELISA: Enzyme-linked immunosorbent assay; FA: Folinic Acid; FCS: Fetal calf serum; HCQ: Hydroxychloroquine; IL: Interleukin; LPS: Lipopolysaccharide; MTT: 3-(4,5-dimethylthiazol-2-yl)-2,5 diphenyl tetrazolium bromide; MTX: Methotrexate; NF-kB: Nuclear factor kappa B; PAR: Parthenolide; RA: Rheumatoid arthritis; ROS: Reactive oxygen species; THEO: Theophylline; TNF: Tumor necrosis factor.

## Competing interests

The authors declare that they have no competing interests.

## Authors’ contributions

NJO designed the experiments, analyzed the data and wrote the manuscript. CFS assisted with data analysis and preparation of the manuscript. TMA analyzed the data and co-wrote the manuscript. All authors read and approved the final manuscript.
